# Supervised Deep Learning Techniques for Image Description: A Systematic Review

**DOI:** 10.3390/e25040553

**Published:** 2023-03-23

**Authors:** Marco López-Sánchez, Betania Hernández-Ocaña, Oscar Chávez-Bosquez, José Hernández-Torruco

**Affiliations:** División Académica de Ciencias y Tecnologías de la Información, Universidad Juárez Autónoma de Tabasco, Cunduacán 86690, Tabasco, Mexico

**Keywords:** image captioning, computer vision, natural language processing, convolutional neural network, recurrent neural network

## Abstract

Automatic image description, also known as image captioning, aims to describe the elements included in an image and their relationships. This task involves two research fields: computer vision and natural language processing; thus, it has received much attention in computer science. In this review paper, we follow the Kitchenham review methodology to present the most relevant approaches to image description methodologies based on deep learning. We focused on works using convolutional neural networks (CNN) to extract the characteristics of images and recurrent neural networks (RNN) for automatic sentence generation. As a result, 53 research articles using the encoder-decoder approach were selected, focusing only on supervised learning. The main contributions of this systematic review are: (i) to describe the most relevant image description papers implementing an encoder-decoder approach from 2014 to 2022 and (ii) to determine the main architectures, datasets, and metrics that have been applied to image description.

## 1. Introduction

The ability to generate automatic descriptions of images connects two fields: computer vision and natural language processing, both of which are outstanding in computer science. Computer vision is needed to extract the characteristics of images, while natural language processing techniques help to convert those characteristics into a proper description for humans. Due to the relationship between both disciplines, the description of images is strongly tied to advances in both fields, finding deep learning as a point of union.

Using deep learning models to generate automatic descriptions of images includes the implementation of convolution neural networks (CNN) to perform the artificial vision part and the extraction of characteristics. Additionally, most of the models based on deep learning use recurrent neural networks (RNN) to perform natural language processing tasks. For this reason, automatic image captioning is currently linked to the set of deep learning techniques and architectures; therefore, this work will focus on these. In addition, other deep learning techniques are used to solve this problem, such as generative adversarial networks (GANs) [1,2,3,4,5]. GANs are an emerging method of semi-supervised and unsupervised learning; therefore, they are not included in this review.

This paper is organized as follows: Section 2 describes the background and related work. Section 3 presents the methodology. Section 4 focuses on the results and discussions regarding the research questions. Finally, Section 5 presents the conclusion.

## 2. Background

In this section, we first outline three deep learning algorithms identified in the literature, introduce the basic concepts of image description, and describe the overall encoder-decoder architecture. This architecture is based on deep learning methods for image description.

### 2.1. Image Description

**Figure 1:**
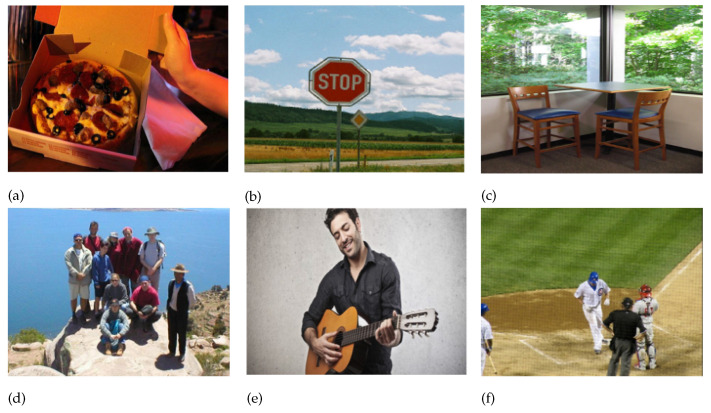
Examples of captions generated by automatic image description models. (**a**) A person holding a box of pizza [8]. (**b**) A stop sign is on a road with a mountain in the background [9]. (**c**) A wooden table and chairs arranged in a room [10]. (**d**) Five people are standing and four are squatting on a brown rock in the foreground [11]. (**e**) A man in a black shirt is playing a guitar [12]. (**f**) A group of baseball players playing a game of baseball [13].

The American Anthropological Association defines *image description* as “a detailed explanation of an image that provides textual access to visual content; most often used for digital graphics online and in digital files” [6]. Automatic image description, also known as image captioning or photo captioning, is the process of generating a concise, human-readable description of the content of an image. This natural language sentence should describe the objects, entities, and relationships as well as people can describe them [7]. Therefore, accurate image description is a challenging task requiring state-of-the-art technology in computer vision and natural language processing. Figure 1 shows a few images and their corresponding automatic captions.

As an emerging issue in deep learning, there are promising applications for automatic image description, such as:

- Alt-text generation for visually impaired people. Blind and low-vision individuals can understand webpage images or real-world scenes by automatically converting an image into text and describing the image using a text-to-speech system. This technique may allow visually impaired people to obtain as much information as possible about the contents of a photograph.
- Content-based image retrieval (CBIR). It consists of recovering a specific subset of pictures (or a single image) from relevant keywords reflecting the visual content found in the image. CBIR and feature extraction approaches are applied in various applications, such as medical image analysis, remote sensing, crime detection, video analysis, military surveillance, and the textile industry.

Currently, the standard approach for automatic image description consists of an encoder-decoder implementation using deep learning techniques to extract and interpret the characteristics of an image in order to generate a sentence describing the scene. Convolutional neural networks (CNN) and recurrent neural networks (RNN) are the usual protagonists of the encoder-decoder approach.

Generally speaking, the fundamental element for neural networks’ functioning is information, measured by entropy. Neural networks try to preserve the most significant amount of input information as it passes between the different layers and compress it to optimize their performance. Different neural network architectures tackle this problem with their strategies, depending on the task they want to perform. For example, convolutional neural networks specialize in image recognition and classification, while recurrent neural networks solve natural language processing tasks, such as text translation or opinion and speech analysis.

### 2.2. Convolutional Neural Networks

Convolutional neural networks (CNN) is a deep learning algorithm specialized in image classification. The core element of CNN is processing data through the convolution operation. In 1990, LeCun et al. [14] published the seminal paper founding the modern framework of a CNN and later improved it in [15]. A CNN specializes in emulating the functionality and behavior of our visual cortex [16].

**Figure 2:**
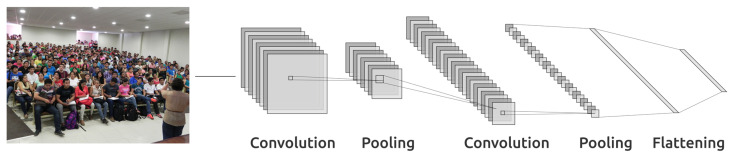
Typical architecture of a CNN.

Figure 2 shows a typical CNN including three types of layers:

- Convolution layer: Feature extraction is carried out through filters called kernels, each generally followed by a ReLU layer.
- Pooling layer: A sweep is applied to obtain statistical information, thus reducing the vector that represents the processed image.
- Flattening layer: Finally, a flattening layer is applied to change the matrix in a one-dimensional vector; the resulting vector will be the one that feeds the neural network to perform detection or classification tasks.

### 2.3. Recurrent Neural Networks

Recurrent neural networks (RNNs) [17] have been widely used in deep learning. RNNs appear as a solution to the problem of information isolation over time. This is a problem for specific data that depend on their predecessors, such as text, because independently analyzing each word causes a loss of important information. To overcome this limitation, RNNs allow backward connections in the network to feed on previously processed information as a kind of memory. RNNs are extensively used in computer vision, particularly in the automatic generation of image descriptions using the encoder-decoder model [18]. In an RNN model, as more layers are added to the neural network, the gradients of the loss function approach zero, making the network hard to train. This is known as the “vanishing gradient” effect.

**Figure 3:**
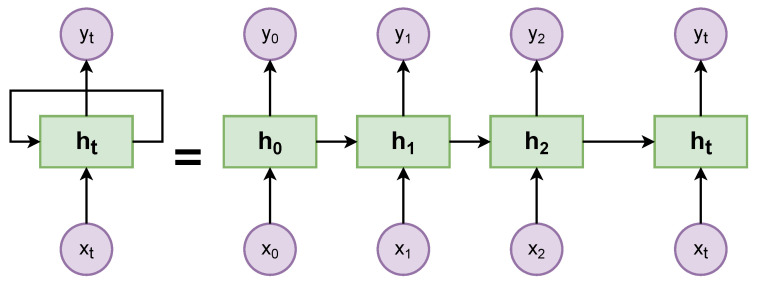
Typical architecture of an RNN.

An RNN simulates a discrete-time dynamical system that has an input $x_{t}$, an output $y_{t}$, and a hidden state $h_{t}$. In Figure 3, the subscript t represents time. Recurrent neural network architecture is a sequence of neural networks that are connected one after the other by backpropagation. Figure 3 illustrates a displaced RNN. On the left side, the RNN is unrolled after the equal sign; the different time-steps are visualized, and information is passed from one time-step to the next [17].

### 2.4. Long Short-Term Memory

**Figure 4:**
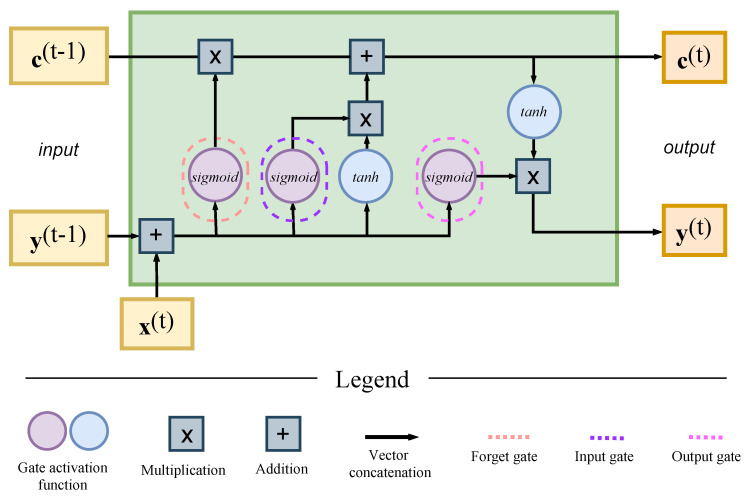
Typical architecture of an LSTM block.

LSTM is a variant of the RNN model proposed to address the vanishing gradient problem. This architecture presents a memory cell that allows for maintaining its state over time, supported by units known as gates. The LSTM setup most commonly used in the literature is called Vanilla LSTM [19]. Figure 4 shows the architecture of a typical vanilla LSTM block.

The major elements of LSTM are [20]:

- Block input: updates the block input component, which combines the current input $x^{\left(t\right)}$ and the output of that LSTM unit $y^{\left(t-1\right)}$ in the last iteration.
- Input gate: combines the current input $x^{\left(t\right)}$, the output of that LSTM unit $y^{\left(t-1\right)}$, and $c^{\left(t-1\right)}$ in the last iteration.
- Forget gate: the LSTM unit determines which information should be removed from its previous cell states $c^{\left(t-1\right)}$.
- Cell: computes the cell value, which combines the block input $z^{\left(t\right)}$, the input gate $i^{\left(t\right)}$, and the forget gate $f^{\left(t\right)}$ values with the previous cell value.
- Output gate: calculates the output gate, which combines the current input $x^{\left(t\right)}$, the output of that LSTM unit $y^{\left(t-1\right)}$, and the cell value $c^{\left(t-1\right)}$ in the last iteration.
- Output block: combines the current cell value $c^{\left(t\right)}$ with the current output gate value.

### 2.5. Encoder-Decoder Approach

The encoder-decoder architecture was born for machine translation. In this architecture, an encoder network encodes a phrase in some languages as a fixed-length vector. Then, another decoder network reads the encoding vector and generates an output sequence in a new language.

**Figure 5:**
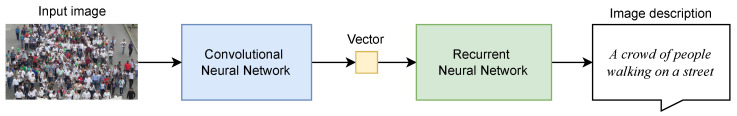
A general architecture for the description of images using deep learning.

First, the encoder-decoder approach uses a deep learning model to encode the image into a characteristics vector. Next, the decoder model uses the input vector to generate a natural language sentence that describes the picture [21]. This is the most common approach to tackling image description, given the promising results for this task. CNNs have been and continue to be the most used network architecture for image encoding and feature extraction. In contrast, RNNs have the function of decoding these characteristics in sentences, i.e., in the description of the image [22]. Both models are trained jointly in the encoder-decoder architecture to maximize the likelihood of the sentence given the image [23]. Figure 5 provides an overview of the basic concepts and the mechanism of an automatic image description generator [7].

A typical encoder-decoder pipeline includes extracting, filtering, and transforming image/caption pairs to obtain an accurate model for automatic image description. The following steps represent a minimal workflow to train models for automatic image description:

1. Select a dataset. It is necessary to use a dataset that includes a large collection of images (in the range of one thousand images), each with several subtitles providing a precise description of its content.
2. Encoder (feature extraction model). CNNs are the de facto tool that extracts the input image features. A CNN performs dimensionality reduction, where the pixels of the image are represented in such a way that interesting parts of the figure are captured effectively into extracted feature signals. Currently, we can take on this task by one of the following:
  - Training the CNN directly on the images in the image captioning dataset;
  - Using a pre-trained image classification model, such as the VGG model, ResNEt50 model, Inception V3, or EfficientNetB7.
3. The extracted feature signals are represented in a fixed-length encoding vector. This fixed-length vector includes a rich representation of the input image.
4. Decoder (language model). RNNs are the de facto tool for working with sequence prediction problems. At this stage, the RNN takes the fixed-length encoding vector and predicts the probability of the next word in a sequence, given a list of words present in that sequence. The output is then the natural language description for the image. Currently, LSTM networks are a commonly used RNN architecture, as they allow for encapsulation of a broader sequence of words or sentences than conventional RNNs.

## 3. Methodology

**Figure 6:**
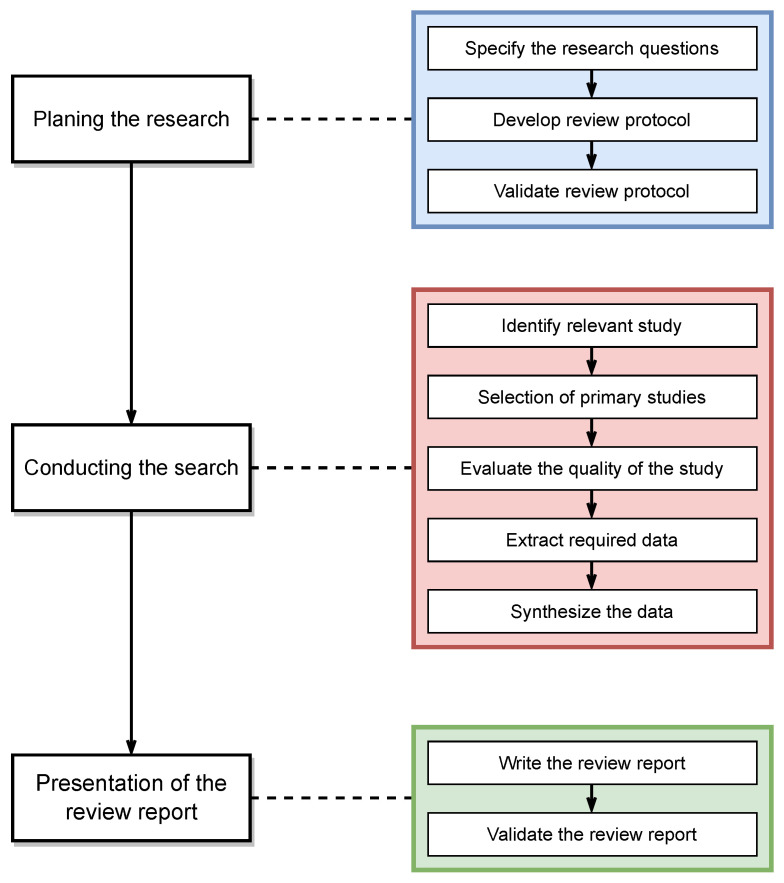
Systematic review process.

The systematic literature review method is based on the methodology proposed by Barbara Kitchenham [24]. Figure 6 shows the systematic review process used in this work, which consists of three phases:

(1) *Planning the research.* The first phase consists of making the appropriate research questions to identify the topic for research. This work examines the following research questions (RQ):
  - RQ1: What is the custom architecture implemented in the encoder-decoder approach used in the research papers?
  - RQ2: Which datasets are used to train and test the models?
  - RQ3: Which metrics were used to evaluate the obtained results?
  These three questions form the basis for developing a research strategy for the literature extraction.
  Following the research question definition, the next activity in the planning phase involves a selection of sources to define the search strategy. For this study, the following international online bibliographic databases were selected:
  - IEEE Xplore.
  - ACM Digital Library.
  - ScienceDirect.
  - Springer.
  The searches were limited to peer-reviewed publications written in English and released between 2014 and 2022. The keywords used for the searches were “Encoder-decoder for automatic image description’,’ “Encoder-decoder for automatic image captioning”, “deep learning for image description”, and “Evaluation of image description generator models”. The last step in the planning phase was selecting and evaluating the research papers. During this phase, an initial selection was made involving the selection of titles, keywords, and abstracts of the possible primary studies.
  This first phase allows us to identify the current view of the research problem, define the latest models and approaches used to solve it, and confirm that it is of current interest.
(2) *Conducting the search*. The second phase regarding the methodology is to perform document extraction and analyses from online databases. The following requirements were applied to ensure that the findings were appropriately classified:
  - The design and implementation of a deep learning model for image description is the central topic that this study proposes.
  - The primary studies report all of the essential components on which an encoder-decoder approach for image description is built.
  - The primary studies report all of the metrics used to evaluate the image description model.
  - The research articles mention the datasets employed.
  To obtain a concise list of articles, a comparison check was conducted to detect duplicate papers. In addition, an analysis of the introduction and conclusion sections was mandatory to know which papers to select or discard. After analyzing and evaluating 91 research articles, 53 were chosen based on their relevance to the topic of study.
(3) *Presentation of the review report*. The final phase of the systematic review framework was to derive analytical results from answering research questions. This is presented in the next section.

## 4. Review and Discussion

**Figure 7:**
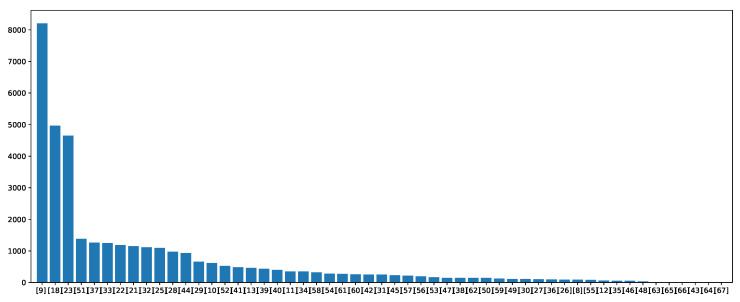
Citations per research article.

**Figure 8:**
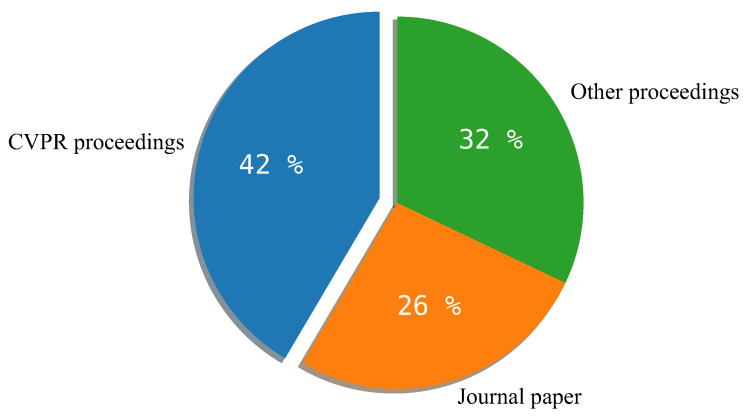
Distribution of research publication sources.

**Figure 9:**
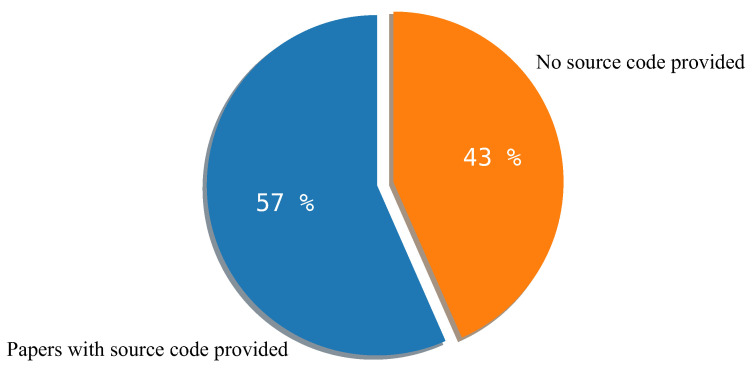
Percentage of papers with source code online.

**Figure 10:**
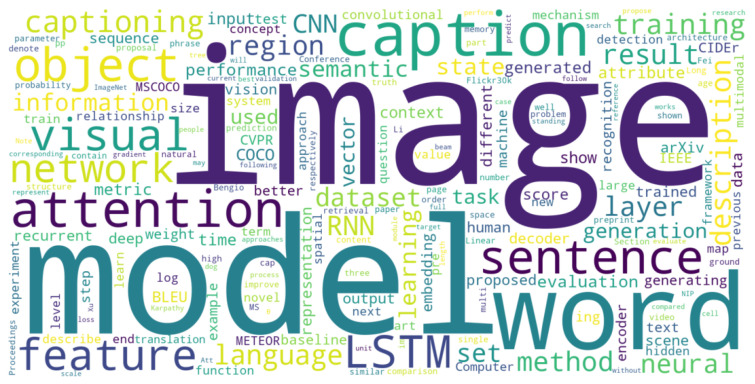
Cloud of words with the most representative terms found in the relevant papers.

According to the systematic literature review, 53 scientific articles were finally selected. The publication dates of the selected papers fall within the time interval of 2014–2022. The number of citations for each of the publications to date is shown in Figure 7. Of these, 42% were published in the proceedings of the IEEE Conference on Computer Vision and Pattern Recognition (CVPR), the reference conference in the field. Additionally, 17 papers (32%) were published in other conference proceedings. Finally, the remaining 14 papers (26%) were published in research journals, as shown in Figure 8. In addition, it was found that 30 of the 53 publications are available on the web for use by the scientific community in GitHub repositories. It is worth mentioning that some works share their model online [9,12,18,21,22,23,25,26,27,28,29,30,31,32,33,34,35,36,37,38,39,40,41,42,43] (as presented in Figure 9), highlighting the use of the Python programming language in all of them and the use of the TensorFlow and PyTorch frameworks. Figure 10 shows the 200 most repeated terms found in the 53 research papers. The terms “image” and “model” are the most repeated words, but “attention”, “LSTM”, and “sentence” are common terms across all papers. Datasets and metrics also appear in the list: “MSCOCO”, “ImageNet”, “BLEU”, and “METEOR”. Additionally, author names, venues, and online repositories are listed: “Karpathy”, “CVPR”, and “arXiv”.

The following subsections are introduced from the data in Table 1 and describe the three architectures that generate automatic descriptions of images.

### 4.1. Main Architectures

- CNN + RNN. In this architecture, a CNN is employed to extract the characteristics of the image, while an RNN is employed to generate the description. A total of 10 works out of 53 (19%) follow this method. It is worth noticing that this architecture is used in the first works on automatic image description using the encoder-decoder approach.
- CNN + LSTM. This architecture uses a CNN encoder and an RNN with LSTM modules to prevent vanishing gradient. Most of the works, including the recent ones (41 out of 53, representing 77%), followed this method.
- CNN + CNN. This architecture uses two CNNs; the first is for extracting characteristics from the image, and the second is for generating the image description from the first CNN results. Only two works (4%) follow this method.

**Figure 11:**
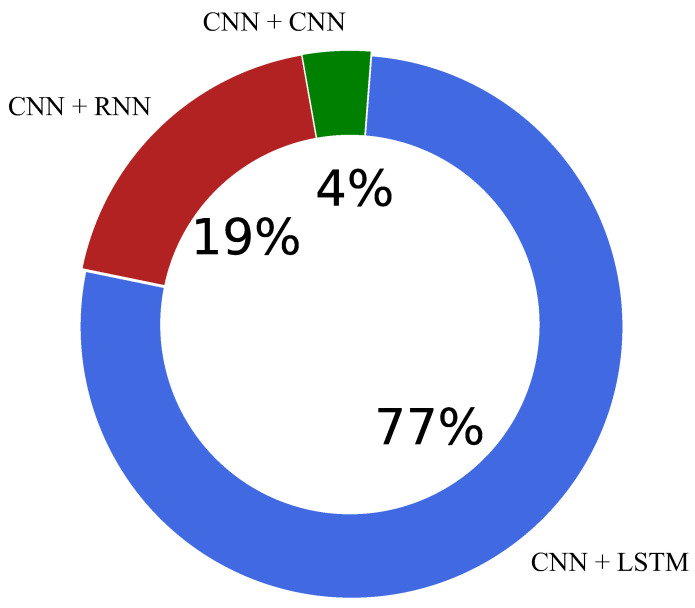
Encoder-decoder architectures’ distribution.

Figure 11 depicts the distribution of the encoder-decoder approaches found in the literature.

According to the literature review, we notice that a CNN is consistently implemented for the encoder module. On the other hand, the decoder module can be implemented using three different architectures: a plain RNN, LSTM, or another CNN. Most of the automatic image description models use an LSTM architecture on the decoder side, and this is due to the effectiveness during the memorization of the data sequence through the memory cells.

### 4.2. CNN + RNN Architecture

The year 2014 can be considered the starting point for automatic image description using deep learning. Karpathy et al. [44] popularized the use of an encoder-decoder approach. The novelty of the encoder-decoder approach was that it was possible to map the images to a fixed set of sentences using deep learning techniques. Following this approach, Mao et al. [11] presented a multimodal RNN (m-RNN) model to generate novel sentence descriptions to explain the content of images. Later, Mao et al. [25] improved their previous work using more complex image representations and more sophisticated language models.

Kiros et al. [10] mapped the features of the image in a shared space with the features of the words in a multimodal space technique. In addition, they improved their approach in [21] by proposing a new neural language model called the structure-content neural language model (SC-NLM). This model allowed a better extraction of the sentence structures, thus improving the generation of captions.

Chen et al. [13] proposed a reverse projection model. It has an additional recurrent layer that performs a reverse projection, allowing for dynamic updating of the visual representations of an image from the generated words. Fang et al. [22] worked on sub-regions of the image instead of the whole image; they used AlexNet and VGG16Net to extract features from the sub-regions.

Karpathy et al. [23] continued their seminal work by proposing a model that uses specific image regions to generate natural language descriptions. Their model used a novel combination of a CNN and an RNN called deep visual–semantic alignments. Following, Yang et al. [45] proposed an image caption system that exploits the parallel structures between images and sentences using a classic RNN.

More recently, Zhou et al. [41] introduced a method using a shared multi-layer transformer network, which can also be adjusted for vision and language generation.

### 4.3. CNN + LSTM Architecture

In 2015, Vinyals et al. [18] introduced the implementation of an LSTM architecture for the decoder module. The encoder network is a CNN, and the decoder network is a stack of LSTM layers. In five works ([36,48,57,58,59]), the authors use LSTM on the decoder side following this same approach.

The following four works present models using the objects and the relationships within the scene to generate the descriptions. Mao et al. [29] proposed a reference expression, which generates a description of a specific object or region. This method allows one to infer the object or region that is being described, thus generating relatively unambiguous sentences. Hendricks et al. [31] proposed a method called the deep compositional captioner (DCC), which can represent the generation of descriptions of objects that are not present in the datasets of paired image sentences. Yao et al. [52] proposed a method that uses a copying mechanism and a separate object recognition dataset, thus managing to generate descriptions of new objects not found in the training sentences. Wang et al. [55] proposed the skeleton key, which first locates the objects and their interactions and then identifies and extracts the relevant attributes to generate the image descriptions. The method decomposes the description into two parts: skeleton sentences and sentences with attributes.

The two following works enrich the description by using information from the faces that appear in the scene. Sugano et al. [46] used the information of the gaze presented in the faces that appeared in the images to enrich the description of the scene. Additionally, Tran et al. [49] introduced a method that can detect a diverse set of visual concepts and generate sentences recognizing celebrities; this method achieved a remarkable performance.

The following seven works employ diverse techniques to improve image description. Mathews et al. [47] proposed a method called SentiCap, which generates descriptions of images with negative or positive feelings. Wu et al. [60] proposed a method to achieve high-level descriptions by implementing a question-guided knowledge selection scheme to rule out the noise information, achieving a better description of the images. Yang et al. [63] proposed a new model, CaptionNet, to help the LSTM avoid the accumulation of errors derived from words irrelevant to generating the image captioning. Zhong et al. [64] proposed a framework to make image captions include specific words and have a better syntactic structure. They also proposed a syntactic dependency structure aware model (SDSAM) to support the framework. Cornia et al. [40] proposed a method called M2, which features a memory mesh transformer for sentence generation. Deng et al. [66] proposed a novel hierarchical memory learning (HML) framework to train with sentences containing thick predicates and sentences containing thin predicates. This gives the resulting sentences more detail in their descriptions of the scene than traditional approaches. More recently, Fei [67] proposed a model that generates descriptions that effectively exploit the global context of the scene without implying an additional cost of inference. The model is trained with two sets: one contains the description labels, and the other includes the description of the general context of the image.

In the following sections, we grouped the remaining CNN + LSTM architecture research papers into three approaches: attention-based image description, semantic-based image description, and reinforcement learning-based image description.

#### 4.3.1. Attention-Based Image Description

The models using attention-based mechanisms focus on prominent regions of an image to generate sentences, considering the scene as a whole.

Xu et al. [9] were the first to introduce an attention-based image description method. The authors developed a richer encoding that allows the decoder to learn where to focus attention in the image while generating each word within the description. Currently, this is the most successful approach for automatic image captioning, and it is also the most cited paper in the collection with over 8000 citations to date (Figure 7). Following the line of attention-based image description, Jin et al. [27] proposed a method capable of extracting information from the scene based on the semantic relationship between textual and visual information. Johnson et al. [28] proposed DenseCap, a method that locates an image’s salient regions and then generates descriptions for each region. Lu et al. [32] proposed a novel adaptive attention model with a visual sentinel; the sentinel helps predict non-visual words such as “the” and “of”. Chen et al. [33] introduced a novel CNN named SCA-CNN that incorporates spatial- and channel-wise attention into a CNN. Pedersoli et al. [53] proposed a method that uses attention mechanisms that associate regions of an image with words from the subtitles given by the decoder. Tavakoli et al. [35] proposed a method of description of images based on attention. This method is based on how humans first describe the most important objects before the less important ones.

Huang et al. [39] proposed a method in which they use a module called “attention on attention” (AoA), allowing them to determine the relevance between the results for attention and consultation. Furthermore, they applied the module (AoA) to their description model’s encoder and decoder. Pan et al. [42] introduced a unified attention block called X-Linear, which allows the network to perform multimodal reasoning to capitalize on visual information selectively. Ding et al. [62] proposed a model that uses two attention mechanisms: stimulus-driven and concept-driven. They introduced the theory of attention in psychology to image caption generation and obtained a good performance. Klein et al. [43] presented a variational autoencoder framework that allows for taking advantage of the region-based image features via an attention mechanism for coherent caption generation. Their experiments demonstrate that this approach generates accurate and diverse captions with varied styles expressed in the image.

#### 4.3.2. Semantic-Based Image Description

Semantic-based image descriptions aim to enrich the language to generate sentences with semantic concepts.

Jia et al. [26] proposed a modification to the LSTM, called the guided LSTM (gLSTM), which allows for generation of long sentences. In this architecture, the network can extract semantic information from each sentence by adding it to each gate and LSTM cell state. Ma et al. [50] proposed a method that generates meaningful semantic descriptions using structural words of the following form: (object, attribute, activity, and scene). Yang et al. [30] proposed a dense subtitle method. This method consists of using the visual characteristics of a region and the subtitles provided for that region combined with the context characteristics and applying an inference mechanism to them, thus achieving a semantically enriched description. Gan et al. [34] developed a semantic composition network (SCN) for image captions, in which the semantic concepts are detected from the image to feed the LSTM network. Venugopalan et al. [38] proposed a method that uses external sources, such as semantic knowledge extracted from unannotated text and labeled images from object recognition datasets. Tian et al. [65] proposed a multi-level semantic context information (MSCI) network. The model updates the different semantic features of an image and then implements a context information extraction network to take advantage of the context information between the semantic layers, thus improving the accuracy of the visual task generation.

It should be noted that two works presented models based on both attention-based and semantic-based mechanisms. You et al. [51] introduced a semantic attention model that allows for a selective focus on the semantic attributes of an image. Liu et al. [56] proposed a method that uses two types of supervised attention models: strong supervision with alignment annotation and weak supervision with semantic labeling. This allows for correction of the attention map at each time-step.

#### 4.3.3. Reinforcement Learning-Based Image Description

Reinforcement learning is a machine learning approach in which an agent aims to discover data and labels through exploration and a reward signal.

Ren et al. [54] introduced a novel method to describe images based on reinforcement learning, which predicts the next best word in the sentence with the help of two networks, the political and value networks. Zhang et al. [8] presented a method based on actor–critic reinforcement learning, which proposes an advantage per token when using LSTM, achieving better descriptions. Rennie et al. [37] proposed a reinforcement-learning-based image description method that generates highly effective descriptions by implementing a time inference algorithm.

### 4.4. CNN + CNN Architecture

Aneja et al. [61] proposed an architecture using only convolutional networks on both the encoder and the decoder sides (they did not use any recursive function). Following this same approach, Wang et al. [12] also used two CNNs; the model was three times faster than the show-and-tell model (which uses LSTM for the decoder module). Both works used a CNN on both the encoder and decoder sides.

### 4.5. Datasets

Most datasets used to train models for the automatic description of images are also used for related tasks, such as face detection. For this reason, some of these datasets also contain classes and bounding boxes in their pictures. The datasets found in the literature were:

- MS COCO [68]. The Microsoft Common Objects in Context (COCO) caption dataset was developed by the Microsoft team; it is aimed at scene understanding. It contains images of complex daily scenes and can perform multiple tasks, such as image recognition, segmentation, and description. The dataset contains 165,482 images and a text file containing nearly one million descriptions. This is the most used dataset, with 77% (41 out of 53) of the revised works using it.
- Flickr8k/Flickr30k [69,70]. The images in the Flickr8k set come from Yahoo’s photo album website, Flickr, which contains 8000 photos. Flickr30k is the extended version of the previous dataset and contains 31,783 images collected from the Flickr website. They usually capture real-world scenes and contain five descriptions for each image. Among the revised works, the Flicker8k dataset occupies second place as it is used in 13 of the 53 works, which is equivalent to 25%. Flickr30k takes third place as it is used in 11 works, which is equivalent to 21%.
- The Visual Genome Dataset [71]. This dataset was created to support research that connects structured image concepts with language. It contains 108,077 images along with 5.4 million region descriptions. Three works use this dataset [28,30,66], corresponding to 6% of the total.
- The IAPR–TC12 Dataset [72]. This dataset has 20,000 images. These are collected from various sources, such as sports, photographs of people, animals, and landscapes. The images in this dataset contain captions in multiple languages. Three works use this dataset [10,11,25], corresponding to 6% of the total.
- The Stock3M Dataset [55]. This dataset has more than 3.2 million images uploaded by users and is 26 times larger than the MS COCO dataset. The images contained in this dataset are very diverse and include people, nature, and made-man objects. Only one work uses this dataset [55] (2% of the total).
- The MIT-Adobe FiveK Dataset [73]. This dataset contains 5000 images. The images correspond to diverse scenes, subjects, and lighting conditions, mainly composed of images of people, nature, and human-made objects. Only one work uses this dataset [49] (2% of the total).
- The SBU Captions Dataset [74]. SBU can be considered an old dataset that contains images and short text descriptions. This dataset is used to induce word embeddings learned from both images and text. This dataset contains one million images with the associated visually relevant captions. Only one work uses this dataset [10], corresponding to 2% of the total.
- The PASCAL Dataset [75]. This dataset provides a standardized image dataset for object class recognition and a common set of tools for accessing the datasets annotations, and it enables the evaluation and comparison of different methods. Since the dataset is an annotation of PASCAL VOC 2010, it has the same statistics as the original dataset. The training and validation sets contain 10,103 images, while the testing set contains 9637 images. Three works [22,35,49] (6%) use this dataset.
- UIUC [76]. This dataset contains eight sports events categories: rowing (250 images), badminton (200 images), polo (182 images), bocce (137 images), snowboarding (190 images), croquet (236 images), sailing (190 images), and rock climbing (194 images). The images are divided into easy and medium according to the human subject judgment. Information on the distance of the foreground objects is also provided for each image. Only one work uses this dataset [50] (2% of the total).
- ImageNet [77]. This dataset consists of 150,000 hand-labeled photographs collected from Flickr and other search engines. Three works [31,37,38] (6%) use this dataset.

**Figure 12:**
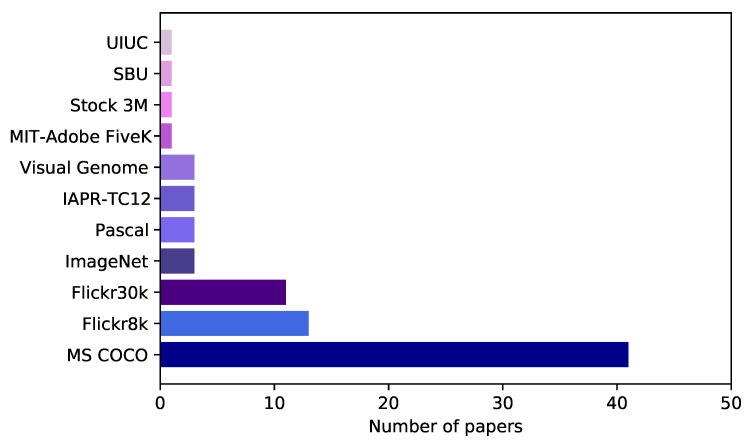
Number of works using each dataset.

Figure 12 presents the use of each dataset in the selected works. MS COCO is the most used dataset, with 41 papers. The vast number of pictures, the quality and resolution of each photograph, and more than one description for each image make it the best dataset to train and test models for image description. It is worth noticing that most of the papers employ two or more datasets in their experiments.

### 4.6. Evaluation Metrics

When evaluating a model based on the quality of the generated language, it is necessary to use particular metrics since traditional metrics, such as accuracy, precision, or recall, cannot be used directly when comparing two texts in natural language. For this reason, image description has used a series of standards, originally from machine translation, to compare descriptions. The evaluation metrics found in the literature were:

- BLEU (Bilingual evaluation understudy) [78]. This is the most widely used metric in practice. The original purpose of this metric is not the image description problem but the machine translation problem. Based on the evaluation of the accuracy rate, it is used to analyze the correlation of n-gram (continuous sequences of words in a document) matches between the system-generated translation and the reference translation statement. It consists of detecting the number of individual words that match. A total of 100% of the papers presented in this review used this metric.
  The BLEU metric first computes the geometric average of the modified n-gram precision, pn, using n-grams up to a length *N* and the positive weights wn plus one. Next, let *c* be the length of the candidate translation, and let *r* the effective length of the reference corpus. Calculate the brevity penalty BP. The BLEU metric ranges from 0 to 1. Few sentences will attain a score of one unless they are identical to the reference sentences:
  (1) $$\mathrm{BP}=\left\{\begin{array}{cc} 1 & \;\mathrm{if}\;c>r\\ e^{\left(1-r/c\right)} & \;\mathrm{if}\;c\leq r \end{array}\right.$$
  then,
  (2) $$\mathrm{BLEU}=\mathrm{BP}\cdot \exp\left(\sum_{n=1}^{N}w_{n}\log p_{n}\right)$$
- ROUGE (Recall-oriented understudy for gisting evaluation) [79]. This is a set of metrics commonly used to evaluate automatic summaries and translation tasks. Again, it is based on comparing n-grams between a hypothesis against one or several references. This metric is used in 33 out of 53 works, which is equivalent to 62%. ROUGE is an n-gram recall between a candidate summary and a set of reference summaries. Formally, ROUGE-L is computed as follows:
  (3) $$\;\mathrm{ROUGE}\;-L=\frac{\left(1+\beta^{2}\right)R_{LCS}P_{LCS}}{R_{LCS}+\beta^{2}P_{LCS}}$$
  when $R_{LCS}=\frac{LCS(C,R)}{c},\;P_{LCS}=\frac{LCS(C,R)}{r},\;\beta =\frac{P_{LCS}}{R_{LCS}}\cdot c$, and r represents the length of the candidate and reference. The higher the ROUGE indicator value, the higher its quality.
- METEOR (Metric for evaluation of translation with explicit ordering) [80]. This is also a metric used to evaluate the result of machine translation. As with BLEU, the basic unit of evaluation is the sentence. The algorithm first creates an alignment between the translation generated from the machine translation model with the reference translation statement. This metric is used in 47 out of 53 works, which is equivalent to 89%.
  The METEOR score for this pairing is computed as follows: Based on the number of mapped n-grams found between the two strings (*m*), the total number of unigrams in the translation (*t*), and the total number of unigrams in the reference (*r*), calculate the precision of the unigram P=m/t and retrieve the unigram R=m/r. Then, calculate a parameterized harmonic mean of *P* and *R*:
  (4) $$F_{\mathrm{mean}\;}=\frac{P\cdot R}{\alpha \cdot P+(1-\alpha )\cdot R}$$
  Meteor computes a penalty for a given alignment, as follows. First, the sequence of matched unigrams between the two strings is divided into the fewest possible number of “chunks” such that the matched unigrams in each chunk are adjacent (in both strings) and in identical word order. The number of chunks (ch) and the number of matches (*m*) is then used to calculate a fragmentation fraction: frag=ch/m. The penalty is then computed as:
  (5) $$Pen=\gamma \cdot frag^{\beta }$$
  The value of γ determines the maximum penalty (0≤γ≤1). The value of β determines the functional relation between fragmentation and the penalty. Finally, the METEOR score for the alignment between the two strings is calculated as follows:
  (6) $$\;\mathrm{score}\;=\left(1-\;\mathrm{Pen}\;\right)\cdot F_{\mathrm{mean}\;}$$
- CIDEr (Consensus-based image description evaluation) [81]. This is a metric especially designed to evaluate image descriptions. All of the words in the description (both candidates and references) are transformed to their respective lemma or root to broaden the search for unigrams to not just exact matches. This metric is used in 15 out of 53 works, which is equivalent to 25%. The CIDEr formula is as follows:
  (7) $${\mathrm{CIDEr}}_{n}\left(c_{i},S_{i}\right)=\frac{1}{m}\sum_{j}\frac{g^{n}\left(c_{i}\right)\cdot g^{n}\left(s_{ij}\right)}{∥g^{n}\left(c_{i}\right)∥∥g^{n}\left(s_{ij}\right)∥}$$
  where *c* represents a candidate caption, *S* represents a set of reference captions, *n* represents an n-gram to be evaluated, *m* represents the number of reference captions, and $g^{n}\left(\cdot \right)$ represents an n-gram-based frequency vector.
- SPICE (Semantic propositional image caption evaluation) [82]. This is used to measure the efficiency of the models in which the photographs’ titles are compared with the objects included. This metric is used in 24 out of 53 works, which is equivalent to 45%.
  The evaluation is as follows: Given a candidate caption *c* and a set of reference captions $S=\left\{s_{1},\ldots ,s_{m}\right\}$ associated with an image, the goal is to compute a score that captures the similarity between *c* and *S*.
  It defines the subtask of parsing captions to scene graphs as follows. Given a set of object classes *C*, a set of relation types *R*, a set of attribute types *A*, and a caption *c*, it parses c to a scene graph:
  (8) G(c)=〈O(c),E(c),K(c)〉
  where O(c)⊆C is the set of objects mentioned in *c*, E(c)⊆O(c)×R×O(c) is the set of hyper-edges representing relations between objects, and K(c)⊆O(c)×A is the set of attributes associated with objects.
  To evaluate the similarity of candidate and reference scene graphs, it defines the function T that returns logical tuples from a scene graph as:
  (9) T(G(c))≜O(c)∪E(c)∪K(c)
  Then, it defines the binary matching operator ⊗ as the function that returns matching tuples in two scene graphs. It then defines the precision *P*, recall *R*, and SPICE as:
  (10) $$P\left(c,S\right)=\frac{\left|T(G(c))⊗T(G(S))\right|}{\left|T(G(c))\right|}$$
  (11) $$R\left(c,S\right)=\frac{\left|T(G(c))⊗T(G(S))\right|}{\left|T(G(S))\right|}$$
  (12) $$\mathrm{SPICE}\left(c,S\right)=F_{1}\left(c,S\right)=\frac{2\cdot P(c,S)\cdot R(c,S)}{P(c,S)+R(c,S)}$$
  Given the F-score, SPICE is simple to understand and easily interpretable as it is bounded between 0 and 1.
- SPIDEr [58]. This metric is the result of combining the properties of CIDEr and SPICE. This combination is made using a gradient method that optimizes a linear combination of both metrics. It was proposed to overcome some problems attributed to the existing metrics. This metric is used in 34 out of 53 works, which is equivalent to 64%.
  SPIDEr uses a policy gradient (PG) method to optimize a linear combination of SPICE and CIDEr directly:
  (13) $$J\left(\theta \right)=\frac{1}{N}\sum_{n=1}^{N}V_{\theta }\left(s_{0}|x^{n},y^{n}\right)$$
  where θ is the model’s parameters, *N* is the number of examples in the training set, x is the image, and y is the set of captions.
- mrank (Matrix rank) [83]. This metric helps to measure the average rank of the correct description for each image, using the median rank among all sentences. This metric is used in 5 out of 53 works, which is equivalent to 9%.
  The mrank metric is used by some authors to evaluate the detection phase of objects performed for their later description. First, the average recall is calculated using the formula:
  First, the intersection over union (IoU) metric is calculated as follows:
  (14) $$\;\mathrm{IoU}\;=\frac{\;\mathrm{area}\;\left(B_{p}\cap B_{gt}\right)}{\mathrm{area}\left(B_{p}\cup B_{gt}\right)}$$
  The IoU is used to measure the accuracy of an object detector, and it is defined as the area of the intersection divided by the area of the union of a predicted bounding box $\left(B_{p}\right)$ and a ground-truth box $\left(B_{gt}\right)$.
  Then, the recall metric is calculated as follows:
  (15) $$\;\mathrm{recall}\;=\frac{TP}{TP+FN}$$
  where TP represents the number of true positives, and FN represents the number of false negatives.
  Then, the average recall (AR) is calculated:
  (16) $$\;\mathrm{AR}\;=2\int_{0.5}^{1}\mathrm{recall}\left(o\right)do$$
  The AR is the recall averaged over all IoUs ∈[0.5,1.0], where the IoU is represented by *o*.
  Finally, calculate the mean of the average recall (mAR) across all *K* classes.
  (17) $$\;\mathrm{mAR}\;=\frac{\sum_{i=1}^{K}AR_{i}}{K}$$
- PPLX (Perplexity) [10]. This metric was proposed by Kiros et al. to evaluate the effectiveness of using pre-trained word embeddings. Solely this work employs this metric [10].
  PPLX is not only used as a measure of performance but also as a link between a text and the additional modality:
  (18) $$\log_{2}C\left(w_{1:n}|x\right)=-\frac{1}{N}\sum_{w_{1:n}}\log_{2}P\left(w_{n}=i|w_{1:n-1},x\right)$$
  where $w_{1:n-1}$ runs through each subsequence of a length n−1, and *N* is the length of the sequence. First, consider retrieving training images from a text query $w_{1:N}$. For each image x in the training set, we compute $C\left(w_{1:N}|x\right)$ and return the images for which $C\left(w_{1:N}|x\right)$ is lowest. Images achieving low perplexity are considered a good match to the query description.

**Figure 13:**
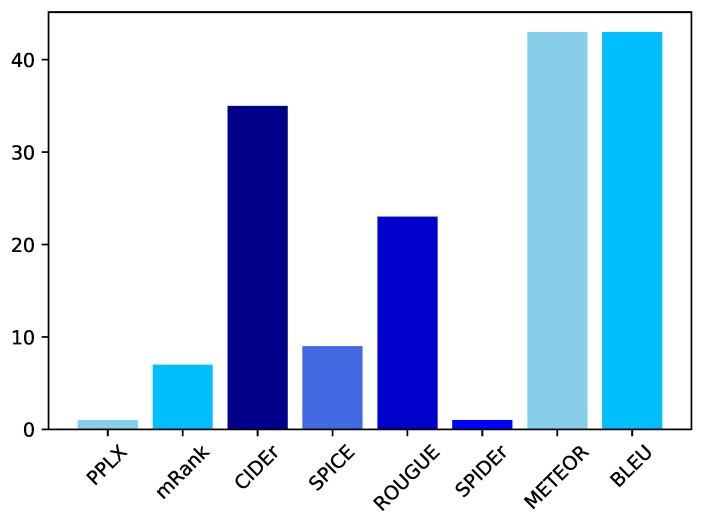
Evaluation metrics usage among all 53 papers.

Figure 13 displays the use of each evaluation metric. BLEU and METEOR stand out as the most used metrics. It is worth mentioning that the BLEU metric is the simplest to use, and no extra configuration is needed. Most of the works reviewed in this article use more than one evaluation metric.

## 5. Conclusions and Future Directions

In this paper, we reviewed and analyzed studies on image description, focusing on encoder-decoder architectures. After analyzing 53 research papers, we conclude that:

- The prevalent architecture for automatic image description employs a CNN as an encoder and an LSTM network as a decoder.
- The most used dataset for training and evaluating models is the MS COCO dataset, used by almost all of the reviewed papers.
- All of the papers in the review use more than one metric to compare the performance of the proposed models, highlighting BLEU and METEOR as the most used metrics.

Based on our study, some research directions for future works concerning automatic image description will focus on the following aspects:

- Multilingual models: The models and advances in the automatic generation of image descriptions have focused solely on the English language. Studying different languages or multilingual datasets would be interesting.
- Amount of data for training: Most of the current models use the supervised learning approach, so they need a large amount of labeled data. For this reason, semi-supervised, unsupervised, and reinforcement learning will be more prevalent in creating future models for generating automatic image descriptions.
- Variety of datasets: The accuracy of the descriptions generated by the existing models depends on the dataset used, and there are few available. It would be interesting to have more and increasingly diverse datasets for future research in this field.

## Figures and Tables

**Table 1:** Selected papers on automatic image description (sorted by date).

Year, Author	Architecture	Datasets	Evaluation Metrics
2014, Karpathy et al. [44]	CNN + RNN	Flickr 8k/Flickr 30K	mRank
2014, Mao et al. [11]	CNN + RNN	Flickr 8k/Flickr 30K, IAPR TC-12	BLEU, mRank
2014, Kiros et al. [10]	CNN + RNN	IAPR TC-12, SBU	BLEU, PPLX
2014, Kiros et al. [21]	CNN + RNN	Flickr 8K, Flickr 30K	mRank
2015, Chen et al. [13]	CNN + RNN	PASCAL, Flickr 8K/30K, MS COCO	BLEU, METEOR, CIDEr
2015, Mao et al. [25]	CNN + RNN	IAPR TC-12, Flickr 8K/ Flickr 30K	BLEU, mRank
2015, Fang et al. [22]	CNN + RNN	PASCAL, MS COCO	BLEU, METEOR
2015, Karpathy et al. [23]	CNN + RNN	Flickr 8K/30K, MS COCO	BLEU, METEOR, CIDEr
2015, Vinyals et al. [18]	CNN + LSTM	Flickr 8K/30K, MS COCO	BLEU, METEOR, CIDEr
2015, Jia et al. [26]	CNN + LSTM	Flickr 8K/30K, MS COCO	BLEU, METEOR, CIDEr
2015, Xu et al. [9]	CNN + LSTM	Flickr 8K/30K, MS COCO	BLEU, METEOR
2015, Jin et al. [27]	CNN + LSTM	Flickr 8K/30K, MS COCO	BLEU, METEOR, ROUGE, CIDEr
2016, Yang et al. [45]	CNN + RNN	MS COCO	BLEU, METEOR, CIDEr
2016, Sugano et at. [46]	CNN + LSTM	MS COCO	BLEU, METEOR, ROUGE, CIDEr
2016, Mathews et al. [47]	CNN + LSTM	MS COCO	BLEU, METEOR, ROUGE, CIDEr
2016, Wang et al. [48]	CNN + LSTM	Flickr 8K/30K, MS COCO	BLEU, mRank
2016, Johnson et al. [28]	CNN + LSTM	Visual Genome	METEOR
2016, Mao et al. [29]	CNN + LSTM	MS COCO	BLEU, METEOR, CIDEr
2016, Tran et al. [49]	CNN + LSTM	MS COCO, MIT-Adobe FiveK	Human Evaluation
2016, Ma et al. [50]	CNN + LSTM	Flickr 8k, UIUC	BLEU, mRank
2016, You et al. [51]	CNN + LSTM	Flickr 30K, MS COCO	BLEU, METEOR, ROUGE, CIDEr
2016, Yang et al. [30]	CNN + LSTM	Visual Genome	METEOR
2016, Anne et al. [31]	CNN + LSTM	MS COCO, ImageNet	BLEU, METEOR
2017, Yao et al. [52]	CNN + LSTM	MS COCO	BLEU, METEOR, ROUGE, CIDEr
2017, Lu et al. [32]	CNN + LSTM	Flickr 30K, MS COCO	BLEU, METEOR, CIDEr
2017, Chen et al. [33]	CNN + LSTM	Flickr 8K/30K, MS COCO	BLEU, METEOR, ROUGE, CIDEr
2017, Gan et al. [34]	CNN + LSTM	Flickr 30K, MS COCO	BLEU, METEOR, CIDEr
2017, Pedersoli et al. [53]	CNN + LSTM	MS COCO	BLEU, METEOR, CIDEr
2017, Ren et al. [54]	CNN + LSTM	MS COCO	BLEU, METEOR, ROUGE, CIDEr
2017, Wang et al. [55]	CNN + LSTM	MS COCO, Stock3M	SPICE, METEOR, ROUGE, CIDEr
2017, Tavakoli et al. [35]	CNN + LSTM	MS COCO, PASCAL	BLEU, METEOR, ROUGE, CIDEr
2017, Liu et al. [56]	CNN + LSTM	Flickr 30K, MS COCO	BLEU, METEOR
2017, Gan et al. [57]	CNN + LSTM	Flickr 30K	BLEU, METEOR, ROUGE, CIDEr
2017, Liu et al. [58]	CNN + LSTM	MS COCO	SPIDEr, Human Evaluation
2017, Gu et al. [36]	CNN + LSTM	Flickr 30K, MS COCO	BLEU, METEOR, CIDEr, SPICE
2017, Yao et al. [59]	CNN + LSTM	MS COCO, ImageNet	METEOR
2017, Rennie et al. [37]	CNN + LSTM	MS COCO	BLEU, METEOR, CIDEr, ROUGE
2017, Venugopalan et al. [38]	CNN + LSTM	MS COCO, ImageNet	METEOR
2017, Zhang et al. [8]	CNN + LSTM	MS COCO	BLEU, METEOR, ROUGE, CIDEr
2018, Wu et al. [60]	CNN + LSTM	Flickr 8K/30K, MS COCO	BLEU, METEOR, CIDEr
2018, Aneja et al. [61]	CNN + CNN	MS COCO	BLEU, METEOR, ROUGE, CIDEr
2018, Wang et al. [12]	CNN + CNN	MS COCO	BLEU, METEOR, ROUGE, CIDEr
2019, Huang et al. [39]	CNN + LSTM	MS COCO	BLEU, METEOR, ROUGE, CIDEr, SPICE
2020, Cornia et al. [40]	CNN + LSTM	MS COCO	BLEU, METEOR, ROUGE, CIDEr, SPICE
2020, Zhou et al. [41]	CNN + RNN	MS COCO, Flick 30K	BLEU, METEOR, CIDEr, SPICE
2020, Ding et al. [62]	CNN + LSTM	MS COCO, Flick 30K	BLEU, METEOR, ROUGE, CIDEr
2020, Pan et al. [42]	CNN + LSTM	MS COCO	BLEU, METEOR, ROUGE, CIDEr, SPICE
2020, Yang et al. [63]	CNN + LSTM	MS COCO, Flick 30K	BLEU, METEOR, ROUGE, CIDEr, SPICE
2021, Zhong et al. [64]	CNN + LSTM	MS COCO, Flick 30K	BLEU, METEOR, ROUGE, CIDEr, SPICE
2021, Tian et al. [65]	CNN + LSTM	MS COCO, Flick 30K	BLEU, METEOR, ROUGE, CIDEr, SPICE
2022, Klein et al. [43]	CNN + LSTM	MS COCO	BLEU, METEOR, ROUGE, CIDEr
2022, Deng et al. [66]	CNN + LSTM	Visual Genome	mRank
2022, Fei [67]	CNN + LSTM	MS COCO	BLEU, METEOR, ROUGE, CIDEr, SPICE

## Data Availability

Not applicable.

## Abbreviations

The following abbreviations are used in this manuscript:

CNN	Convolutional Neural Network
RNN	Recurrent Neural Network
GAN	Generative Adversarial Network
MS COCO	Microsoft Common Objects in Context
LSTM	Long Short-Term Memory
BLEU	Bilingual Evaluation Understudy
RQ	Research Questions
IEEE	Institute of Electrical and Electronics Engineers
ACM	Association for Computing Machinery
ROUGE	Recall-Oriented Understudy for Gisting Evaluation
METEOR	Metric for Evaluation of Translation with Explicit Ordering
CIDEr	Consensus-based Image Description Evaluation
SPICE	Semantic Propositional Image Caption Evaluation
PPLX	Perplexity
IoU	Intersection Over Union
MRANK	Matrix Rank
CBIR	Content-Based Image Retrieval
